# Proximal guided hybrid federated learning approach with parameter efficient adaptive intelligence for pneumonia diagnosis

**DOI:** 10.1038/s41598-025-32286-2

**Published:** 2025-12-21

**Authors:** Keerthika P, Suresh P, Nitesh Kumar AR

**Affiliations:** School of Computer Science and Engineering , Vellore Institute of Technology , Vellore, India

**Keywords:** Data privacy, Vision transformer, Low-Rank adaptation, Medical imaging, Explainable AI, Computational biology and bioinformatics, Engineering, Health care, Mathematics and computing, Medical research

## Abstract

Pneumonia remains a serious worldwide health concern, particularly in low resource countries, where prompt diagnosis is challenging. Early detection relies on chest radiography, but data privacy rules and patient data fragmentation make AI model building difficult. Federated Learning allows collaborative model training without patient data sharing, a promising solution. Standard federated learning methods like FedAvg suffer with data heterogeneity and significant communication overhead. To overcome these constraints, this research proposes an upgraded federated framework with FedProx, which mitigates client drift in non-IID contexts by proximal optimization and Low-Rank Adaptation, a parameter-efficient fine-tuning technique that minimizes communication costs. Vision Transformers are used as the backbone architecture for chest X-ray categorization because they capture the global visual context better than convolutional models. The tiny memory footprint proposed in this research, fits resource-constrained medical infrastructure. The proposed technique was validated for a pneumonia classification job utilizing the publicly available Chest X-Ray Images dataset, which was distributed across simulated clients to replicate real-world healthcare organizations. The model’s performance is measured using accuracy, precision, recall, F1-score, AUC and system-level measures including communication cost per round and convergence rate. The proposed federated model had 88.5% classification accuracy under data heterogeneity and reduced communication overhead and computation cost. Explainability research employing attention heatmaps supports the model’s clinically important pulmonary areas, boosting clinical adoption, trust and transparency.

## Introduction

Pneumonia represents a significant contributor to global morbidity and mortality, especially in children under five and the elderly, resulting in over 2.5 million deaths each year^1^. The most accessible and cost-effective pneumonia diagnosis tool is chest radiography (CXR). However, manual chest X-ray interpretation is laborious and subject to observer variability. Deep learning models, especially CNNs, can automate pneumonia detection with excellent accuracy^2^. Artificial Intelligence (AI) models in medical imaging show promise, however most solutions require centralized learning and patient data aggregation at a single server^3^. This raises concerns about data privacy, security, and compliance, particularly with HIPAA and GDPR. Centralizing data is often impossible due to institutional restrictions, technical constraints, and legal barriers to hospital or national data exchange. Deep learning has improved medical imaging applications beyond the detection of pneumonia. Hassan et al. proposed a deep learning-based approach for early black fungus identification using optimal CNN architectures^4^. The accuracy of their approach and the ability of deep models to identify minor pathological signs support automated fungal infection identification in clinical imaging. Hassan et al. developed an optimized ensemble deep learning model for breast cancer diagnosis using transfer learning and Grey Wolf Optimization (GWO) to improve feature extraction and model convergence^5^. This hybrid approach outperformed the single-model CNN systems in terms of accuracy and generalization. These studies demonstrate the increasing use of deep learning with optimization and ensemble methodologies to address medical image analysis issues, such as class imbalance, interpretability, and feature redundancy. Their findings support the wider use of deep learning-driven diagnostic systems and lay the groundwork for applying hybrid and optimization-based paradigms to complicated diseases, such as pneumonia, in federated learning frameworks.

Federated Learning (FL) is a decentralized training paradigm that lets several institutions (clients) train a global model while protecting their local data. FL requires each client to train its dataset locally and send only model updates to a central server for aggregation^6^. Data privacy and information leakage are protected while healthcare institutions collaborate. FL keeps data private but adds technical obstacles. FL settings often contain non-identically distributed (non-IID) data, which causes client drift, slower convergence, and poor global model generalization^7^.

 FL can be used to segment brain tumors, identify diabetic retinopathy, and diagnose COVID-19 in medical imaging[8]. This research demonstrates FL’s capacity to maintain patient privacy and establish multi-institutional models. Most of these solutions use CNNs and presume that client data distributions are similar. Moreover, deep models typically require the transmission of a large number of parameters, which can strain bandwidth-limited clients. To address this, Low-Rank Adaptation (LoRA) has gained attention for enabling efficient fine-tuning by decomposing model weight updates into low-rank matrices, drastically reducing communication and memory costs^9^. Additionally, Vision Transformers (ViTs) have recently demonstrated superior performance in medical image analysis tasks, including CXR classification, by capturing long-range dependencies and global contexts more effectively than CNNs^10^. However, their high parameter count makes them ideal candidates for parameter-efficient training methods, such as LoRA in FL settings.

This work aims to:

- Develop a privacy-preserving pneumonia detection model using Vision Transformers within a FL framework.
- To improve the model robustness, communication efficiency, and personalization under non-IID data distributions by integrating FedProx and LoRA into the training process.

The key contributions of this research are as follows:

1. A federated architecture that incorporates FedProx and LoRA for the efficient and stable training of a ViT model across distributed medical institutions.
2. Extensive evaluation on a real-world pneumonia dataset (Chest X-Ray Images) under non-IID settings.
3. A detailed analysis of the model performance, communication efficiency, and convergence behaviour demonstrated the advantages of the proposed approach in heterogeneous federated environments.

## Related works

Deep learning, especially CNNs, is used for medical image classification, including pneumonia identification^11^. Rajpurkar et al. presented CheXNet, a 121-layer DenseNet trained on the NIH ChestX-ray14 dataset, which detected pneumonia similar to radiologists^12^. Irvin et al. created CheXpert, a larger labelled dataset with uncertainty labels, for more reliable and scalable deep learning investigations in thoracic imaging^13^. Transfer learning is commonly used in medical imaging to overcome the shortage of labelled data. CNN architectures, including ResNet, VGG, and EfficientNet, pre-trained on ImageNet, have improved the performance and training time on chest X-ray datasets^14^. These models are generally trained on centralized data, which compromises patient privacy and limits their deployment across health care organizations. FL is a new decentralized learning paradigm that allows collaborative model training without sharing raw data. McMahan et al. introduced FedAvg^15^. FL clients train models privately on their private data and update a central server that updates the global network.

 The heterogeneity of real-world healthcare data limits the application of FL^16^. Patient populations, imaging instruments, and annotation processes vary between institutions, resulting in non-IID data, model convergence instability, and performance degradation. FedProx  [^17^] is an extension of FedAvg that penalizes deviation from the global model by adding a proximal component to the local loss function. Local updates are stabilized by this regularization, particularly in heterogeneous data contexts. FedProx improves convergence and robustness in healthcare research, such as federated skin lesion categorization. It is typically used with CNNs and huge parameter counts, decreasing communication efficiency[^17^].

Large models have made parameter-efficient fine-tuning possible via Low-Rank Adaptation (LoRA). To fine-tune with fewer parameters, LoRA injects trainable low-rank matrices into pretrained weight layers instead of updating the complete model^19^. LoRA is utilized in natural language processing (GPT, BERT) and Vision Transformers (ViTs) for picture categorization and segmentation. LoRA reduces communication in distributed or federated situations by transmitting only low-rank matrices between the client and server. LoRA has not been extensively used in federated medical image analysis, notably with FedProx and ViTs, despite its benefits^20^.

Transformers, which were originally intended for NLP, have advanced computer vision. Vision Transformers exploit self-attention mechanisms to outperform CNNs in various vision tasks with sufficient data. In medical imaging, ViTs have performed well in pneumonia identification, lesion segmentation and organ categorization^21^. The global reach and scalability of ViTs make them a promising backbone for FL frameworks. However, their large parameter sizes hinder implementation in low-resource environments and FL communication efficiency. These are useful for LoRA-based fine-tuning in federated contexts. FL, FedProx, LoRA, and ViTs have shown promise individually, but no research has combined them for pneumonia detection. LoRA is rarely used in medical federated situations, and FL research mostly utilizes CNNs and ignores parameter-efficient fine-tuning. This research proposes a federated system that blends FedProx, LoRA, and ViTs to address privacy, personalization, and efficiency issues in pneumonia prediction from chest X-ray images. in FL situations, particularly in non-IID data and communication-constrained applications.

Recent advances have stressed parameter-efficient fine-tuning in medical vision models. LoRA adapters can minimize the amount of trainable parameters while preserving or enhancing chest X-ray performance, improving NIH ChestX-ray14 AUROC by 2.9%. Also promising are ensemble and collaborative learning methodologies. An ensemble federated model employing DenseNet and ResNet architectures for pneumonia detection by Mabrouk et al. achieved good accuracy in a decentralized setup^18^. Another study examined self-supervised ViT pre-training in multi-demographic FL. They show that domain heterogeneity is a problem and recommend pretrained models for non-IID scenarios. Additionally, ViTs are used for severity scoring^22^. A lightweight transformer is proposed for pneumonia severity regression, suggesting classification models can produce more clinically useful outputs. These findings support the proposed design of ViT, FedProx, and LoRA to balance diagnostic performance, communication efficiency, and convergence stability in federated medical contexts^23^.

Recent discoveries show that parameter-efficient fine-tuning in medical vision models works. LoRA adapters sustain or increase chest X-ray performance with minimal configurable parameters. Asokan et al. shows that LoRA can be effortlessly integrated into federated setups like SAM-based 3D segmentation, saving communication^16^. Other improvements have allowed LoRA to adapt foundation models to heterogeneous devices^24^. Medical imaging uses a lightweight LoRA-ViT model with 0.05% trainable parameters to classify cervical cancer. PConv-enhanced LoRA for defect segmentation shows its visual domain flexibility. FL variations of LoRA include SLoRA, FeDeRA and privacy-preserving modifications. FedMS uses sparsely activated foundation models for communication-efficient FL^25^. Cho et al. used NeurIPS model-heterogeneous LoRA techniques to address device heterogeneity^26^.

## Materials and methods

### Problem formulation

This research aims to create a robust and privacy-preserving deep learning system for chest radiograph pneumonia identification. Formally, the task is binary image classification.

#### Input

Chest X-ray picture $\:X\in\:{R}^{H\times\:W\times\:C}$ where H, W and C reflect image height, width, and channel count which is usually grayscale or RGB.

#### Output

A label $\:y\:\in\:\{0,\:1\}$ where 0 indicates no pneumonia and 1 denotes pneumonia.

#### Dataset

Each client (hospital) has a dataset $\:{D}_{i}={\left\{\left({x}_{j},{y}_{j}\right)\right\}}_{j=1}^{{n}_{i}}$ with local chest X-ray images and labels.

### Proposed federated learning framework

**Fig. 1:**
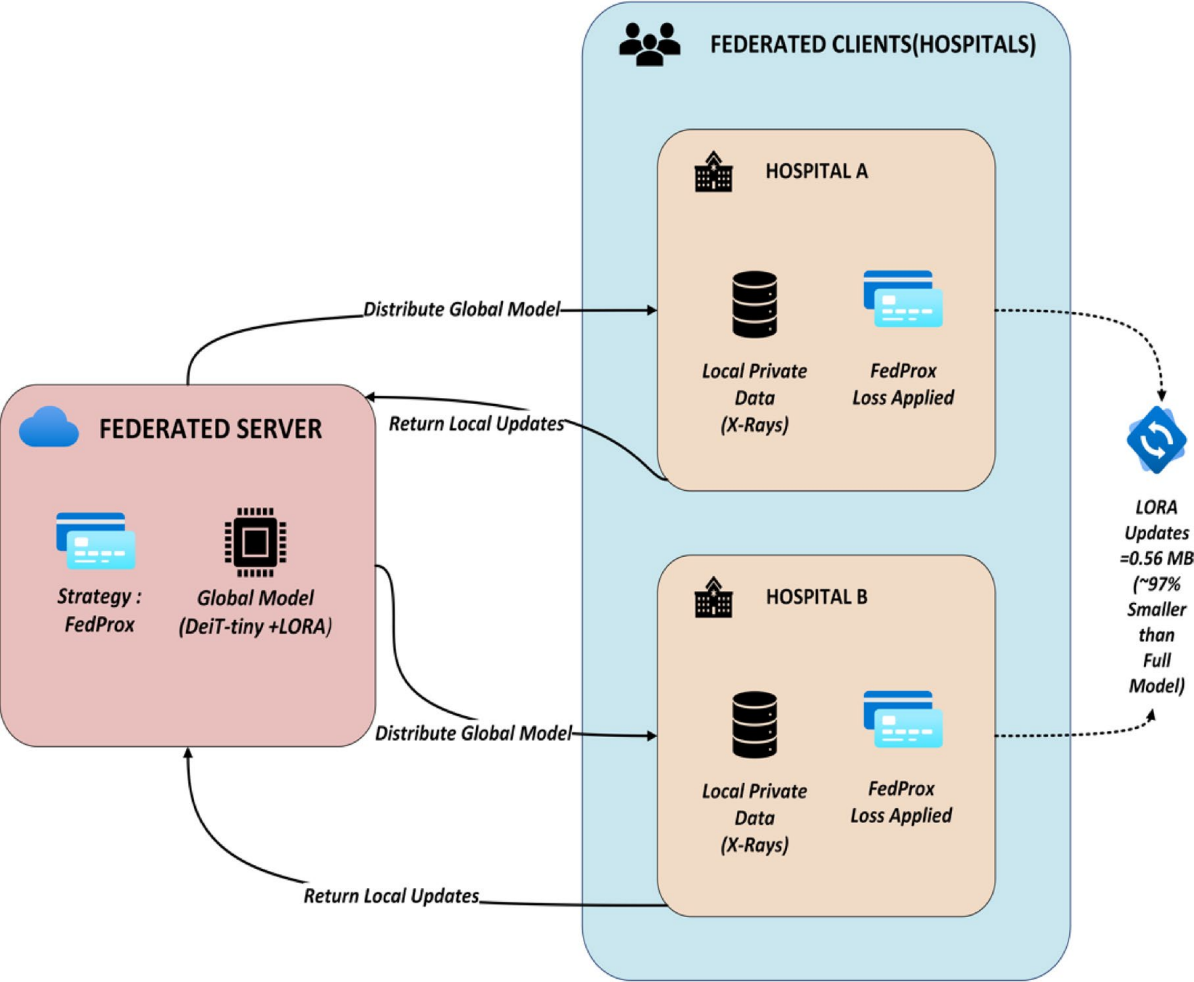
Proposed federated learning setup.

Traditional machine learning requires transferring confidential patient data to a central server for training. HIPAA and GDPR restrict data transfers in medical contexts due to privacy and ethical issues. FL allows many hospitals (clients) to train a global model without sharing raw patient data to solve healthcare data privacy issues. Only model updates are sent between clients and the central server. The individual components of the setup are participating clients, local computation for privacy preservation and a federated server that takes care of orchestration, aggregation, communication efficiency and iterative process. The proposed FedProx-based FL method with LoRA-efficient updates enabled lightweight, privacy-preserving pneumonia detection in this investigation. As shown in Fig. 1, multiple hospitals (federated clients) participates in the training. Pneumonia annotated chest X-ray pictures are stored locally by each facility. Clients include urban centres, rural clinics, and other hospitals in various regions. Patients, imaging equipment, and demographics may vary greatly between these institutions (data imbalance, label skew, feature diversity). This system relies on communication, local training, and global aggregation.

**Fig. 2:**
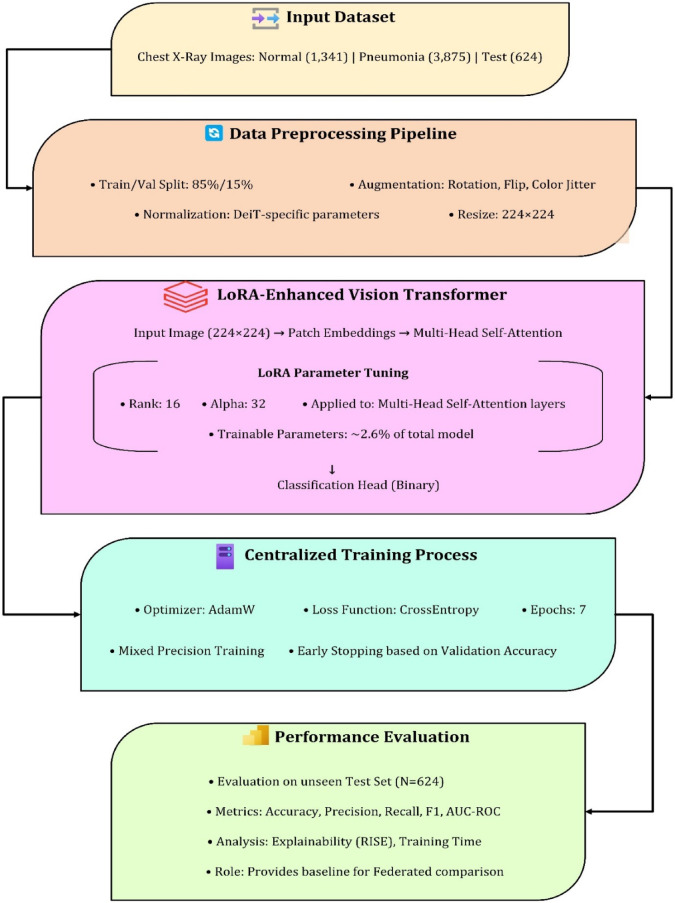
Proposed Model Architecture and Training Flow.

Figure 2 shows the planned centralized pneumonia classification workflow. The technique starts with 1,341 normal and 3,875 pneumonia chest X-ray images and 624 test samples. Images undergo a common preparation pipeline, including 224 × 224 pixel scaling, data augmentation (rotation, flipping, color jitter), and DeiT-specific normalization for the Vision Transformer backbone. A LoRA-enhanced Vision Transformer applies low-rank adaptations (LoRA) to the multi-head self-attention layers in the model architecture. At rank 16 and alpha 32, just 2.6% of model parameters are trained, lowering training overhead and maintaining performance. In the training phase, the AdamW optimizer and CrossEntropy loss function optimize convergence over 7 epochs with mixed precision training and early stopping.

Finally, the trained model is assessed on the held-out test set using accuracy, precision, recall, F1-score, and AUC-ROC. RISE measures explainability, and training duration provide a performance benchmark for federated learning experiments. The procedure in Fig. 2 underpins this study’s federated adaptations.

#### Local client training

**Fig. 3:**
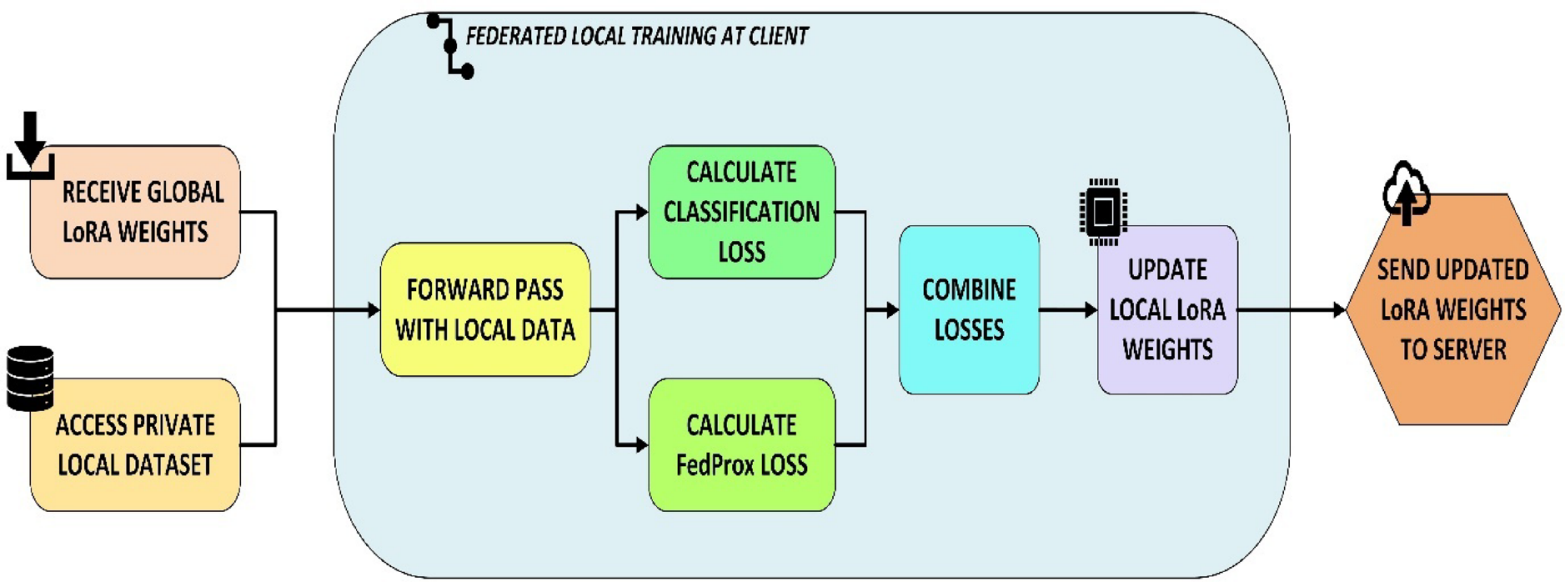
Federated Local Training at Client.

Each client hospital computes and protects privacy locally. Clients receive the global model from the central server and train it locally using their own datasets as shown in Fig. 3. The FedProx loss function given in Eq. 1 stabilizes training on heterogeneous (non-IID) data by adding a proximal term to FedAvg.

Instead of raw data, the model updates are sent to the central server. Updating LoRA modules with efficient parameters reduces transmission size to 0.56 MB. Patient data stays private and on-premises, legal compliance with data protection standards is maintained, and domain adaptation occurs naturally as each client learns from their data. To handle data heterogeneity, each client’s local loss function is modified with a proximal term, which penalizes deviations from the global model.

1 $$\:{L}_{\mathrm{FedProx}}={L}_{\mathrm{local}}+\frac{{\upmu\:}}{2}|w-{w}_{\mathrm{global}}{|}^{2}$$

where $\:{L}_{\mathrm{local}}$ is the standard cross entropy classification loss, $\:w$ are the local weights, $\:{w}_{\mathrm{global}}$ ​ are the current global weights and $\:{\upmu\:}$ is the regularization coefficient. This keeps local models close to the global model, which is significant because hospital patient populations and imaging equipment fluctuate.

#### Global aggregation at server

**Fig. 4:**
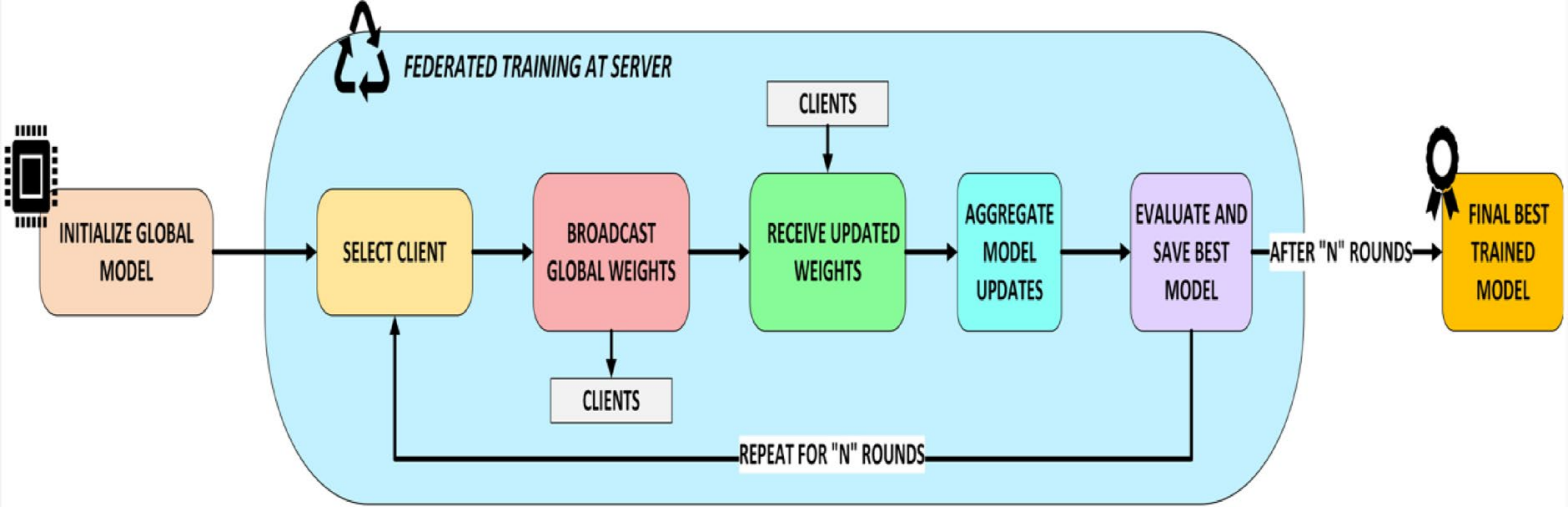
Federated global training at server.

The federated server aggregates local updates from participating clients to update the global model as shown in Fig. 4. The FedProx server considers proximal regularization and other client-specific criteria like data quantity and update quality, not just weights. Federated server orchestrates and aggregates and coordinated federated processes. The server utilizes a weighted averaging strategy to aggregate the LoRA-based updates received from the clients, creating the new global model for the next round. The aggregation comprises two steps such as model initialization and update aggregation. The aggregation process is given by Eq. 2.

##### Model initialization

Starts with a base model DeiT-tiny (Data-efficient Image Transformer) with LoRA and distributes current model to all or chosen hospitals.

##### Update aggregation

Utilizes FedProx approach to aggregate LoRA-based updates, punishing deviations from the global model to handle data heterogeneity.

2 $$\:{w}_{\mathrm{global}}^{t+1}={\sum\:}_{i=1}^{K}\frac{{n}_{i}}{n}{w}_{i}^{t}$$

where $\:{w}_{i}^{t}$ is the LoRA update from client $\:i$, $\:{n}_{i}$ ​ is the number of data samples at client $\:i$ and $\:n={\sum\:}_{i=1}^{K}{n}_{i}$ is the total number of samples from all selected clients. Aggregation updates the model and sends it to all clients for the next communication round.

#### LoRA-integrated efficiency

The communication overhead is a major FL bottleneck. This overhead is overcome with the introduced Low-Rank Adaptation, which fine-tunes just low-rank matrices and they are only trained and transmitted in the DeiT-tiny transformer model to substantially minimize update size. LoRA lowers communication round bandwidth. It allows scalable FL, which is useful in healthcare with edge devices and network constraints. The tiny memory footprint fits resource-constrained medical infrastructure. This reduces update size (~ 97% lower than entire model) and permits deployment in bandwidth-limited contexts. An iterative process is repeated over numerous communication rounds until the global model converges or meets a performance criterion. Each round enhances model generalization without compromising data privacy.

A communication round is a complete FL cycle, including model dissemination, local training, and update aggregation. This cycle continues until the global model converges. Communication rounds greatly impact convergence and model performance. Communication is expensive, especially in low-bandwidth healthcare settings. LoRA reduces communication overhead by up to 97% by compressing updates to ~ 0.56 MB at each round compared to full model transfers.

### BackBone model: vision transformer

**Fig. 5:**
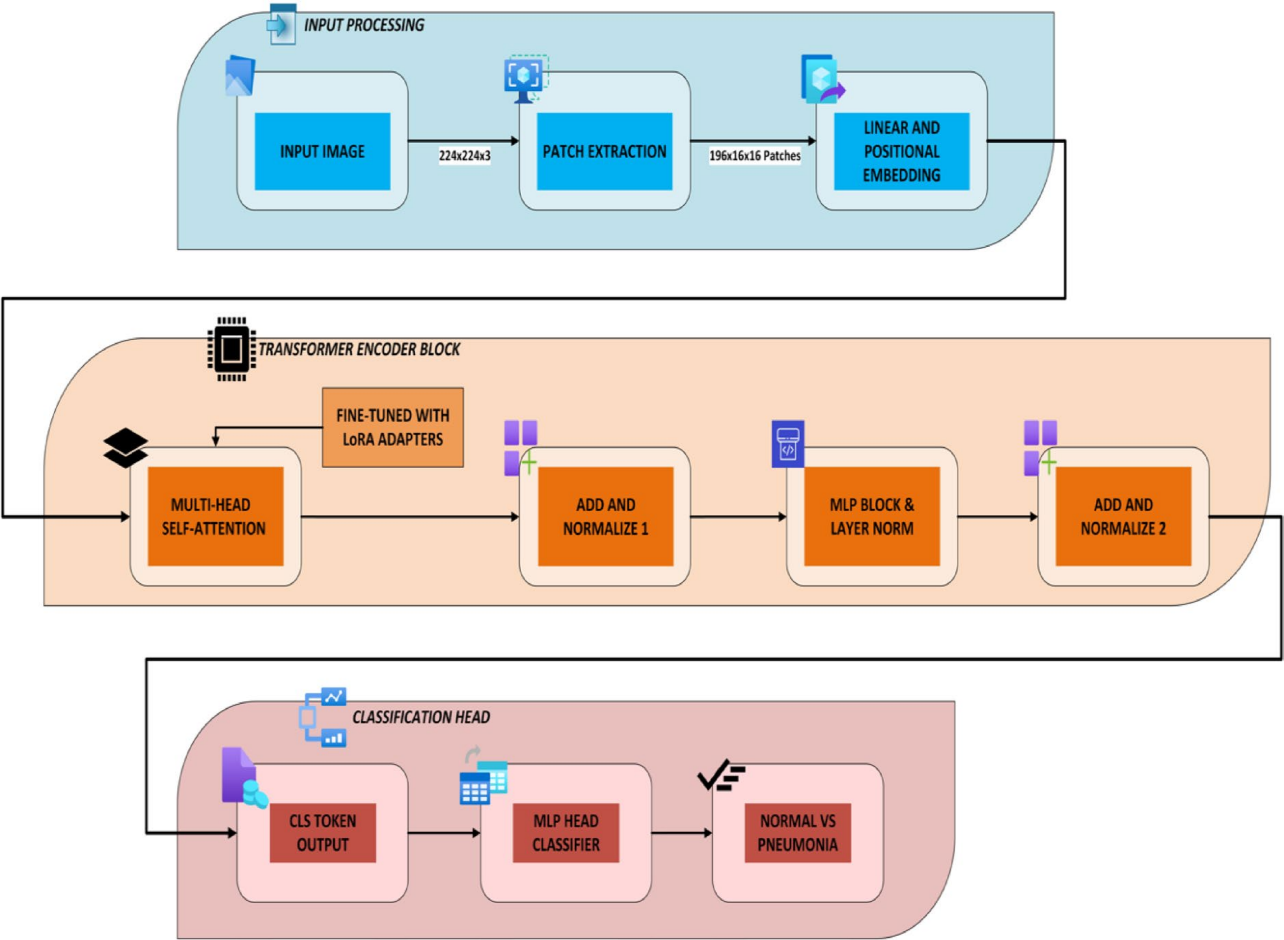
Proposed Backbone model (ViT - DeiT-tiny + LoRA).

The proposed pneumonia classification model uses a Vision Transformer (ViT) backbone for efficiency and communication restrictions in FL. Figure 5 shows the architecture of the proposed model that describes a flow with Input Processing, Transformer Encoder Block, and Classification Head. In this research, this ViT model is enhanced with LoRA for parameter-efficient client device fine-tuning.

#### Input processing block

A chest X-ray image with 224 × 224 × 3 dimension is used as input for the model. The input passes through the following steps.

- **Patch Extraction**: Divide input image into non-overlapping 16 × 16 patches, yielding 196 patches.
- **Linear and Positional Embedding**: Flatten and pass each patch via a learnable linear projection. A learnable positional encoding is provided to retain spatial information lost during flattening as in Eq. 3.

3 $$\:{Z}_{0}=\left[{x}_{cls};{x}_{p}^{1}E;{x}_{p}^{2}E;\dots\:;{x}_{p}^{196}E\right]+{E}_{pos}$$

where $\:{x}_{cls}$ is the class token, $\:{x}_{p}^{i}$ is the i^th^ patch, $\:E$ is the patch embedding matrix and $\:{E}_{pos}$ is the positional encoding.

#### Transformer encoder block

Each embedded input goes through a Transformer Encoder Block stack. One encoder block is shown in the diagram that includes the following.

##### Multi-Head Self-Attention (MHSA)

Calculates paired attention across all tokens. This lets the model capture X-ray image global dependencies.

##### LoRA adapters

LoRA modules are inserted into MHSA query and value projections to reduce training overhead in federated settings. Local training only updates these adapters’ trainable low-rank matrices.

##### Add & normalize 1

Apply a residual connection between MHSA input and output (plus LoRA), then normalize layers.

##### Norm for MLP blocks and layers

A feed-forward network (MLP) with GELU activation is applied to each token separately, followed by residual connection and normalization.

##### Add & normalize 2

An additional skip connection guarantees steady gradients and deep training compatibility.

The DeiT-tiny model learns complex, hierarchical representations from X-ray patches by repeating this encoder block 12 times.

#### Classification head

The classification token (CLS) represents the image’s global context after encoding.

**CLS Token Output** is extracted after the final transformer block.

**MLP Head Classifier** uses a tiny multi-layer perceptron to process the CLS token.

**Prediction Output** which the classifier produces in a binary prediction (0 → Normal and 1 → Pneumonia).

Adding FedProx Loss minimizes client drift in heterogeneous (non-IID) environments, keeps local updates closer to global targets, and improves federated training stability and convergence.

### FedProx algorithm

A standard optimization method in FL is FedAvg which trains clients locally and weighted averages their model changes on the central server. Due to institutional variances in technology, demography, and clinical practices, FedAvg struggles with non-IID data situations, which are common in healthcare. This causes client drift and global model convergence issues. FedProx (Federated Proximal), a robust optimization technique that explicitly handles system and statistical heterogeneity by altering the client-side objective with a proximal term is used[^17^]. A typical FL goal is to minimize a global objective given in Eq. 4.

4 $$\:\underset{w}{\mathrm{min}}f\left(w\right)={\sum\:}_{k=1}^{K}\frac{{n}_{k}}{n}{F}_{k}\left(w\right)$$

where.

- $\:K$ is the number of clients.
- $\:{n}_{k}$ is the number of local samples on client $\:k$.
- $\:n={\sum\:}_{k=1}^{K}{n}_{k}$ is the total number of samples from all selected clients.
- $\:{F}_{k}\left(w\right)=\frac{1}{{n}_{k}}{\sum\:}_{i=1}^{{n}_{k}}\mathcal{\:}\mathcal{l}\mathcal{\:}\left(w;{x}_{i},{y}_{i}\right)$ is the local empirical risk on client $\:k$.
- $\:\mathcal{l}\mathcal{\:}\left(w;{x}_{i},{y}_{i}\right)$ is the loss function for sample $\:\left({x}_{i},{y}_{i}\right)$

FedProx penalizes substantial deviations from global model parameters $\:{w}_{t}$ by adding a proximal component to the local objective function given in Eq. 5.

5 $$\:\underset{w}{\mathrm{min}}{F}_{k}\left(w\right)+\frac{{\upmu\:}}{2}|w-{w}_{t}{|}^{2}$$

where $\:\mu\:$ $\:\ge\:$ 0 is the proximal term coefficient controlling the strength of the regularization, $\:{w}_{t}$ is the global model distributed at communication round $\:t$ and $\:|\:\cdot\:|$ denotes the $\:{\mathcal{l}}_{2}$ norm.

The soft restriction of this proximal term keeps the local model $\:w$ near to the global model $\:{w}_{t\:\:}$, reducing divergence during local updates. Fedprox converges and addresses non-IID client drift. Different data distributions might cause local updates to drift in non-IID data settings. Client drift degrades aggregated models and slows convergence. FedProx controls local deviation so that the proximal term reduces local model divergence from the global model. FedProx ensures more consistent and predictable updates even when local training epochs or data distributions change greatly. It lets clients conduct incomplete optimization due to restricted computation and the algorithm still converges. FedProx excels in heterogeneous medical environments where institutions have different computing and data capabilities.

FedProx has been found to converge with non-IID data and partial client participation under modest assumptions and thereby improve communication efficiency by producing relevant updates in fewer rounds. It also addresses system heterogeneity, where hardware limitations may impede local updates for some clients. The proposed pneumonia prediction method uses FedProx to maintain model consistency across hospitals with skewed X-ray datasets. LoRA integration enhances FedProx by lowering communication overhead and ensuring convergence stability.

### LoRA integration

When using deep models like ViTs or Convolutional Neural Networks (CNNs), FL communication cost is heavily influenced by model update size. This is crucial in healthcare, where hospitals may have limited bandwidth and computational resources. Parameter-efficient fine-tuning approach Low-Rank Adaptation (LoRA) is used to reduce model update size without compromising accuracy. LoRA injects trainable low-rank matrices into weight layers while freezing weights to fine-tune pre-trained deep models.

To update a pre-trained weight matrix $\:{\:W}_{0}\in\:{R}^{d\times\:k}$, instead of updating $\:{\:W}_{0}$directly, LoRA approximates it by multiplying two smaller matrices as in Eq. 6 and it is modified as Eq. 7.

6 $$\:{\Delta\:}W=AB,\hspace{1em}\mathrm{where\:}A\in\:{R}^{d\times\:r},B\in\:{R}^{r\times\:k},\hspace{1em}r\ll\:\mathrm{min}\left(d,k\right)$$

7 $$\:W={W}_{0}+{\upalpha\:}\cdot\:{\Delta\:}W={W}_{0}+{\upalpha\:}\cdot\:AB$$

where.

- $\:r$ is the adaptation matrix rank (set to 16).
- α is the scaling factor (set to 32) that controls update magnitude.

During training, the original pre-trained weights $\:{W}_{0}$ are kept frozen, and only the parameters of the new, low-rank matrices A and B are updated. The number of trainable parameters and model updates provided to the server are greatly reduced.

#### LoRA with vision Transformers

For pneumonia identification from chest X-rays, the proposed design uses the DeiT-tiny global model backbone. LoRA is included into DeiT’s transformer blocks self-attention layers, particularly in parameter-heavy query (Q) and value (V) projections, enabling efficient ViT model fine-tuning at client hospitals. LoRA selectively alters a small portion of model weights during training, resulting in lightweight updates without affecting transformer expressive power. Low-rank adaptation to fully connected layers or large convolutional kernels can extend LoRA to CNN-based architectures, which was first proposed for transformers. The algorithm for federated training with LoRA and FedProx is given in the algorithm 1.

**Algorithm 1:**
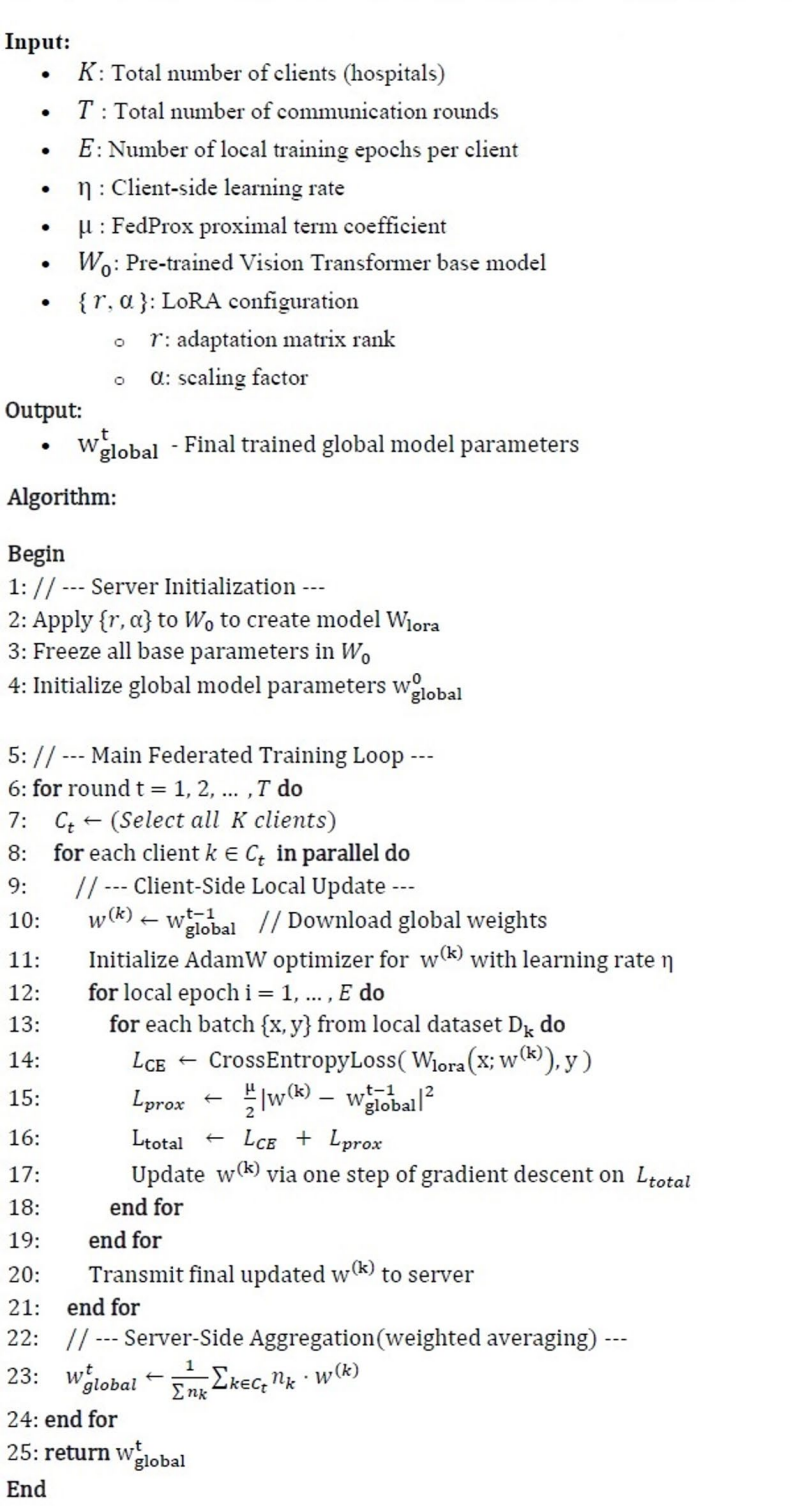
Federated Training with LoRA and FedProx (FedLoRA-Prox).

## Experimental setup

### Dataset description

Kermany et al.‘s Kaggle Chest X-Ray Images (Pneumonia) dataset is used for this research. The dataset includes 5,856 labelled chest radiographs classed as Normal or Pneumonia. The pneumonia class encompasses bacterial and viral illnesses. The dataset is frequently used in medical image analysis and can be utilized to train pneumonia detection deep learning models. The dataset comprises training, validation, and testing directories. Data is processed and partitioned for FL simulations. To guarantee homogeneity and maximize model performance, following preprocessing activities are performed. To comply with the ViT backbone concept, chest X-ray images are scaled to 224 × 224 pixels. Pixel values are scaled to [0.485, 0.456, 0.406] and standard deviation of [0.229, 0.224, 0.225] using standard ImageNet parameters to normalize images. To make the X-rays compatible with pretrained ViT models, they are converted from grayscale to RGB and duplicated across three RGB channels.

**Table 1:** Original data.

Split	NORMAL	PNEUMONIA	Total
Train	1341	3875	5216
Validation	8	8	16
Test	234	3905	624
Total	1583	4273	5856

**Table 2:** Redistributed data.

Split	Images	Percentage Split
Training	4447	75.9%
Validation	785	13.4%
Test	624	10.7%
Total	5856	100.0%

Federation-based data partitioning is performed where simulation of client-specific data partitions replicates the operational situation of several hospitals or medical institutions in FL. 10 clients represent independent hospital nodes in the dataset. The data distribution Non-Independent and identically distributed (Non-IID) is examined. The Non-IID scenario simulates hospital-specific disease prevalence by partitioning the dataset with biased label distributions for each client. Some clients may obtain primarily Pneumonia photos, while others may receive mostly Normal cases. Tables 1 and 2 depicts the original and redistributed data respectively.

**Fig. 6:**
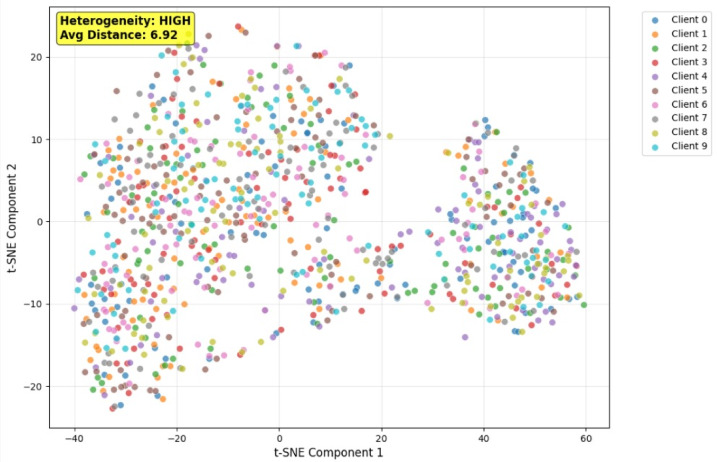
t-SNE Client Data (Non-IID data) Heterogeneity.

Figure 6 shows the client data from the Chest X-Ray Pneumonia dataset visualized using t-SNE. This t-SNE plot shows the data heterogeneity in the Chest X-Ray Pneumonia dataset in a federated non-IID scenario, highlighting the significance of adaptive learning algorithms in medical federated learning applications. The map shows the feature space distribution over 10 clients in a federated learning environment, with each point representing an X-ray image embedding and the colors representing clients 0–9. The distributed and partially segregated color clusters show the non-IID characteristics of dataset partitioning. Client feature representations show large variability in local data distributions, with some clients having more pneumonia instances and others having normal chest X-ray images. The annotation “Heterogeneity: HIGH” with a 6.92 average inter-client distance quantifies this distributional discrepancy. High heterogeneity causes non-uniformity of client data, making federated model convergence and generalization difficult. The apparent divergence between clusters suggests that independent local models trained on biased local feature spaces may diverge, requiring heterogeneity-aware optimization techniques for a steady global performance.

**Table 3:** Hyper-parameters of proposed model.

Category	Hyperparameter	Value
Optimization & Regularization	Optimizer	AdamW
	Learning Rate	1 × 10⁻⁴
	Weight Decay	0.01
	Loss Function	CrossEntropyLoss
	Scheduler	CosineAnnealingLR
	FedProx (µ)	0.5
	Client Fraction	1.0
Parameter-Efficient Fine-Tuning (LoRA)	LoRA Rank (r)	16
	LoRA Alpha	32
	LoRA Dropout	0.1
	Target Modules	query, value
	Trainable Parameters	147,456 (2.6% of total)
	Parameter Reduction	97.40%

Table 3 lists the important hyperparameters of the proposed hybrid Federated Learning framework for Pneumonia Diagnosis. AdamW optimizer, conservative learning rate, weight decay and cosine annealing scheduler offer consistent convergence, generalization and client drift reduction across varied medical datasets. LoRA-based fine-tuning parameters (Rank = 16, Alpha = 32, Dropout = 0.1) minimized the number of trainable parameters by over 97%, enabling efficient communication and computing scalability in federated contexts without compromising diagnostic accuracy.

### Evaluation metrics

Classification performance measures and FL efficiency metrics were used to evaluate the proposed federated pneumonia detection model using ViT with FedProx and LoRA.

#### Classification performance metrics

The global model’s diagnostic efficacy is assessed using these standard metrics:

##### Accuracy

Percentage of accurately predicted incidences across all samples. Accuracy alone may not reflect imbalanced dataset performance, despite its popularity.

8 $$\:\mathrm{Accuracy}=\frac{TP+TN}{TP+TN+FP+FN}$$

##### Precision

Percentage of true pneumonia forecasts compared to all positive predictions.

9 $$\:\mathrm{Precision}=\frac{TP}{TP+FP}$$

##### Sensitivity (Recall)

Measures the accuracy of identifying genuine pneumonia patients.

10 $$\:\mathrm{Recall}=\frac{TP}{TP+FN}$$

##### F1 score

The F1-Score balances false positives and negatives by equalizing precision and recall.

11 $$\:\mathrm{F1-Score}=2\times\:\frac{\mathrm{Precision}\times\:\mathrm{Recall}}{\mathrm{Precision}+\mathrm{Recall}}$$

##### AUC (Area under the ROC Curve)

Measures model’s class distinction across thresholds. Higher AUC means better discrimination.

To assess global model generalization, these metrics are calculated on the central test set using updates from all clients.

#### Federation-based learning efficiency metrics

Federated training efficiency in addition to model performance is evaluated with the metrics communication cost and convergence rate.

**Communication Cost (MB/Round)**:

Total data sent between servers and clients per round. This covers model parameter sizes and gradient exchanges. LoRA-enhanced ViT models communicate only low-rank adapter weights, decreasing bandwidth compared to full model updates.

12 $$\:\text{Communication Cost}=\frac{\text{Total parameters transmitted}\times\:\text{Bit precision}}{8\times\:{10}^{6}}\text{ (in MB)}$$

##### Convergence rate

The number of communication rounds needed to achieve a specific performance level (e.g., 90% accuracy or plateaued validation loss). Learning is more efficient with faster convergence. This is analysed using global loss curves and validation accuracy trends.

## Results and discussion

**Fig. 7:**
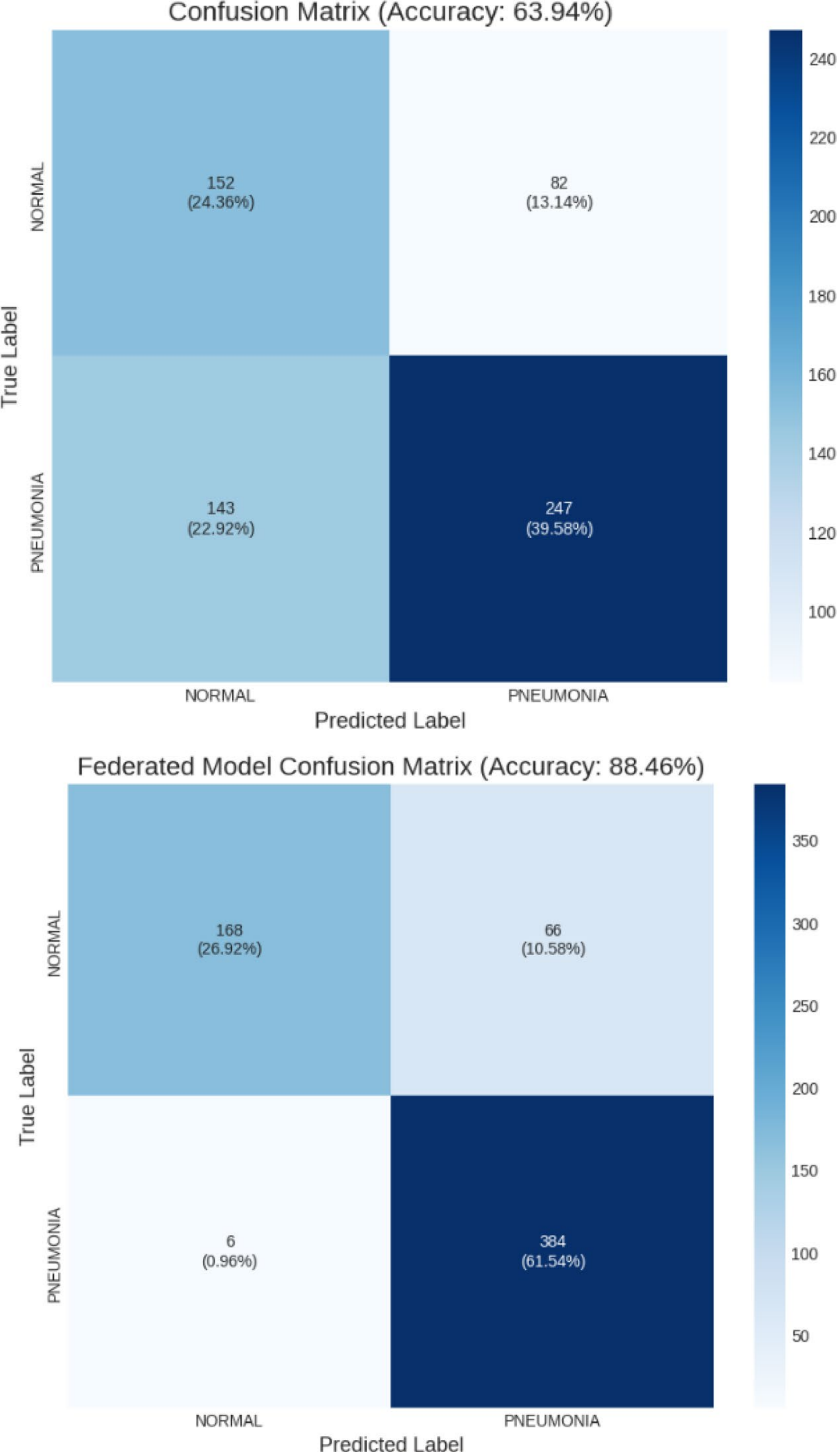
Confusion matrix (**a**). Centralized Model (**b**) Proposed Federated Model.

This section thoroughly evaluates the proposed federated pneumonia detection model employing ViT optimized with FedProx and Low-Rank Adaptation. The results aim to emphasize both the diagnostic performance and operational efficiency of the model under actual FL situations. The discussion begins by comparing the performance of a centralized baseline model with the proposed federated approach, shown through the confusion matrices in Fig. 7a and b. These indicate how efficiently each model distinguishes between pneumonia and normal cases, offering insight into the categorization strengths and flaws.

The confusion matrix shows the centralized model’s inadequate generalization on the unseen test set, resulting in 63.94% accuracy. Model predictions were inaccurate, with 82 false positives and 143 false negatives. The model missed nearly 36% of pneumonia cases due to its high false-negative rate, which is particularly concerning. This indicates that the model overfits to its training data and is unreliable on other test data distributions. Confusion matrix for federated model shows strong diagnostic performance 88.46% accuracy. The model missed only 6 pneumonia cases out of 390, making it a great screening tool. With a 98.46% recall for PNEUMONIA, the model is reliable in detecting the condition. The model’s main drawback is 66 false positives, which is typical for a high-sensitivity diagnostic tool.

**Fig. 8:**
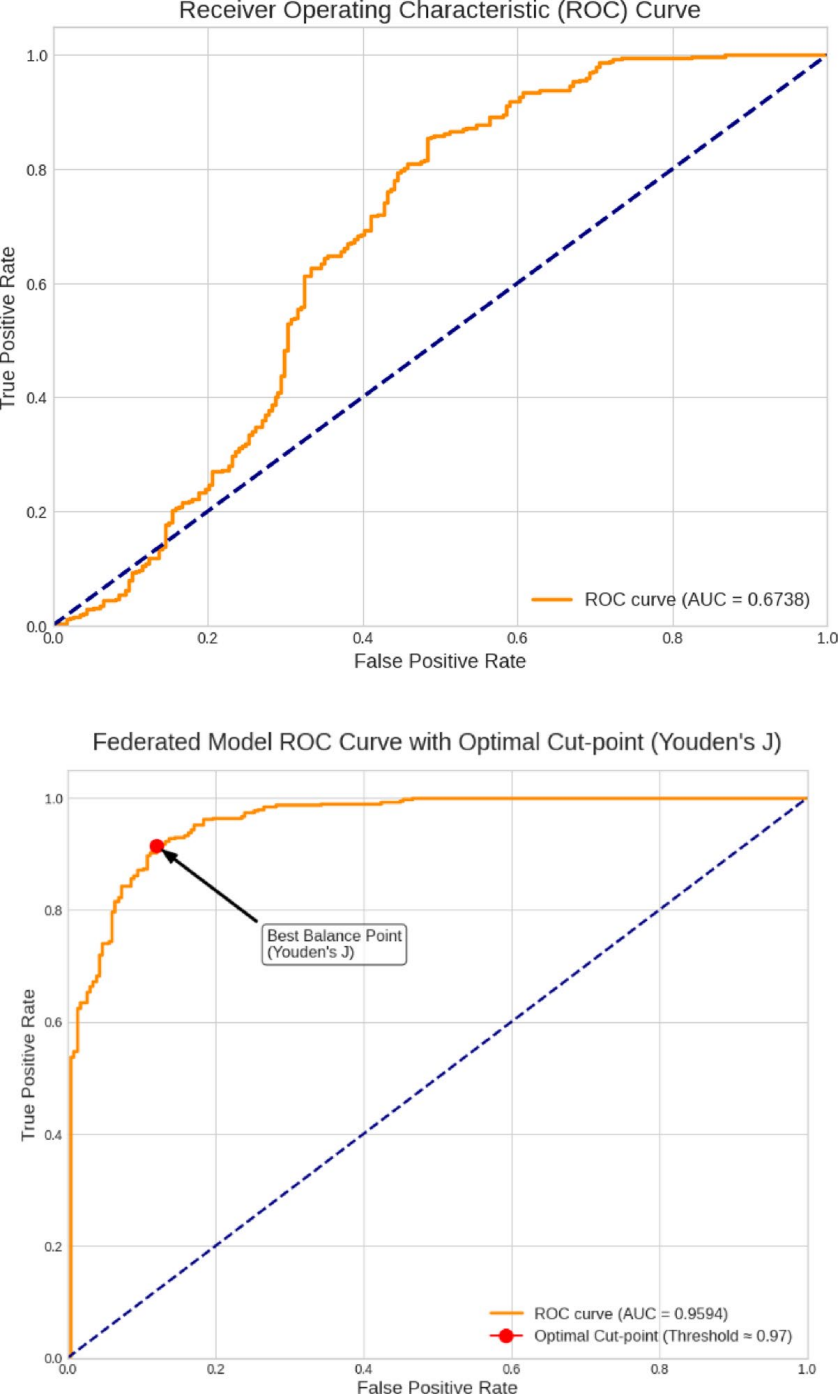
ROC Curve with Optimal cut point.

**Fig. 9:**
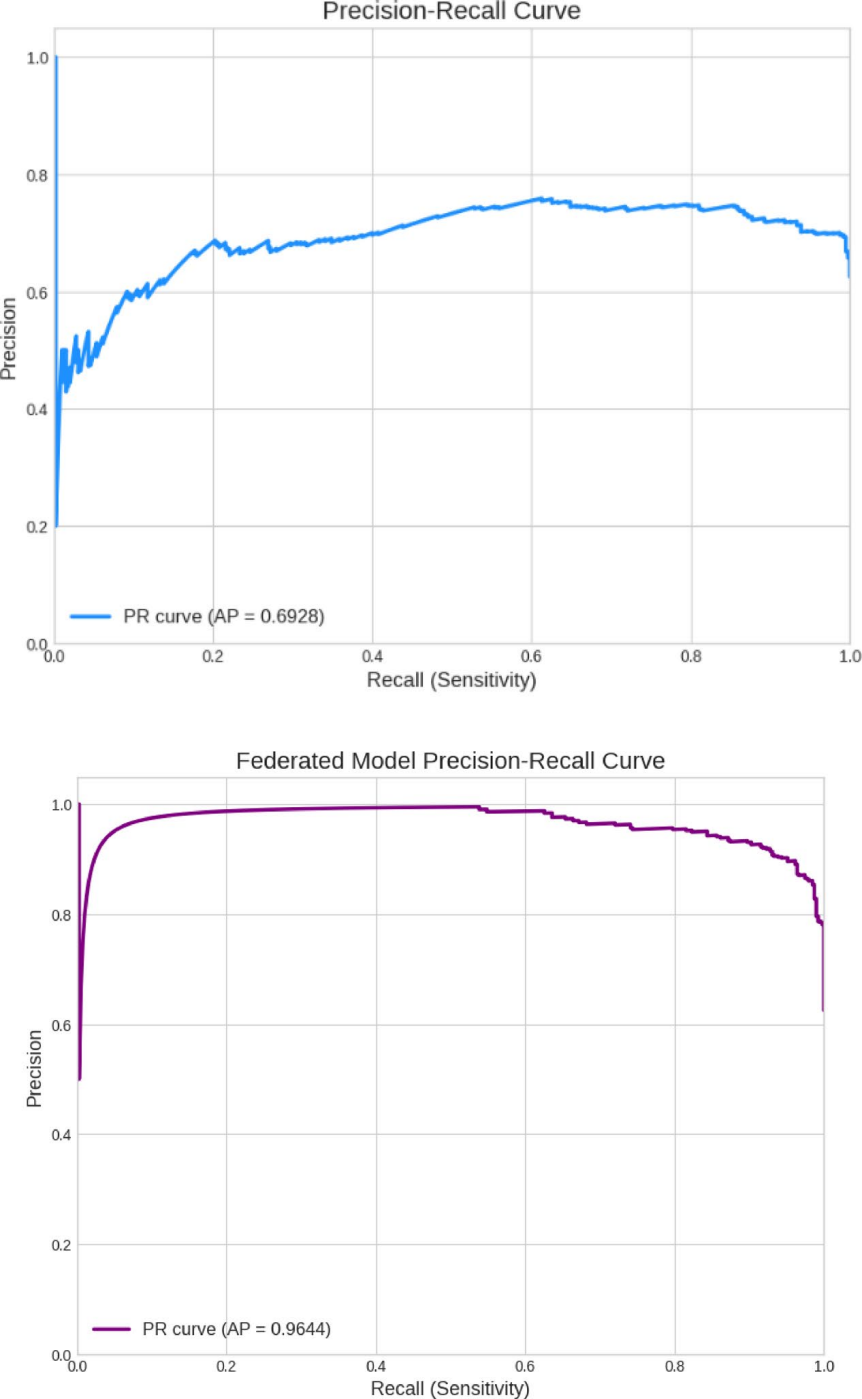
Precision-Recall (PR) Curve.

To further examine classification performance, a Receiver Operating Characteristic (ROC) curve is utilized in Fig. 8 together with the ideal threshold cut-point and the Precision-Recall (PR) curve depicted in Fig. 9, which are particularly essential for evaluating models under class imbalance scenarios. Both employ AUC to summarize performance. The centralized model’s ROC curve shows its low discrimination, with an AUC of 0.6738. This result, while better than random chance (AUC = 0.5), shows that the model struggles to differentiate ‘NORMAL’ from ‘PNEUMONIA’ situations. The curve’s proximity to the diagonal line supports the conclusion that the model overfit to the training data’s distribution and has poor test set generalization. The federated model’s ROC curve has a high Area Under the Curve (AUC) of 0.9594, indicating good discrimination. This score, near 1.0, shows the model’s ability to discriminate ‘NORMAL’ and ‘PNEUMONIA’ situations. The analysis also finds an ideal decision threshold of 0.97 using Youden’s J statistic. This cut-point strikes the optimal compromise between sensitivity and specificity, demonstrating the model’s clinical applicability.

The centralized model’s Precision-Recall (PR) curve has an Average Precision (AP) score of 0.6928, indicating poor pneumonia case identification. As the model searches for more true positives (recall), precision reduces significantly, as shown by the curve. This result matches the low AUC score and shows that the centralized model is not a robust classifier on unknown test data, exposing its failure to generalize.

The Precision-Recall (PR) curve for the federated model shows its great performance with an Average Precision (AP) score of 0.9644. The curve, which remains high during recall, shows that the model can detect most pneumonia cases without losing precision. This shows that the model’s positive predictions are accurate, proving its diagnostic power and test set generalization. Table 4 compares centralized and federated settings using accuracy, precision, F1-score and recall. It is inferred that the Federated Model outperformed the Centralized Model on the unseen test set. The Federated Model’s accuracy was 88.46%, surpassing the Centralized Model’s poor result by over 24% points. The Federated Model’s 98.46% recall, far better than the centralized model’s 63.33%, detected nearly all genuine pneumonia cases. The Federated Model’s 96.55% accuracy in recognizing NORMAL instances indicates its reliability in classifying healthy patients.

**Table 4:** Performance based on accuracy, precision, F1 score and recall.

Class	Metric	Centralized Model	Federated Model (Superior)
PNEUMONIA	Recall (Sensitivity)	63.33%	98.46%
	Precision	75.08%	85.33%
	F1-Score	68.71%	91.43%
NORMAL	Recall (Specificity)	64.96%	71.79%
	Precision	51.53%	96.55%
	F1-Score	57.47%	82.35%
Overall	Accuracy	63.94%	88.46%

Table 5 shows the communication cost per round, showing how LoRA adapters reduce communication overhead. LoRA in the federated framework dramatically improves communication efficiency, which is essential for system rollout. Table 2 shows how the method decreases trainable parameters from 5.6 million to 147,456. This reduces the communication payload per client in each training round by 97.40%, from 21.64 MB to 0.56 MB. For real-world clinical settings with restricted network capacity, the suggested federated system is efficient and scalable due to its low communication overhead.

**Table 5:** Trainable parameters and communication Payload.

Metric	Standard Fine-Tuning (Centralized Model)	Proposed Approach (LoRA)	Reduction (%)
Trainable Parameters	5,672,258	147,456	97.40
Communication Payload *(per client*,* per round)*	21.64 MB	0.56 MB	97.40

The computational efficiency is claimed in terms of training time for the proposed federated model in Table 6.

**Table 6:** Training Time.

Models	Training Time (sec)
Standard Fine-Tuning (Centralized Model)	1833
Proposed Federated Approach (LoRA)	1107

**Fig. 10:**
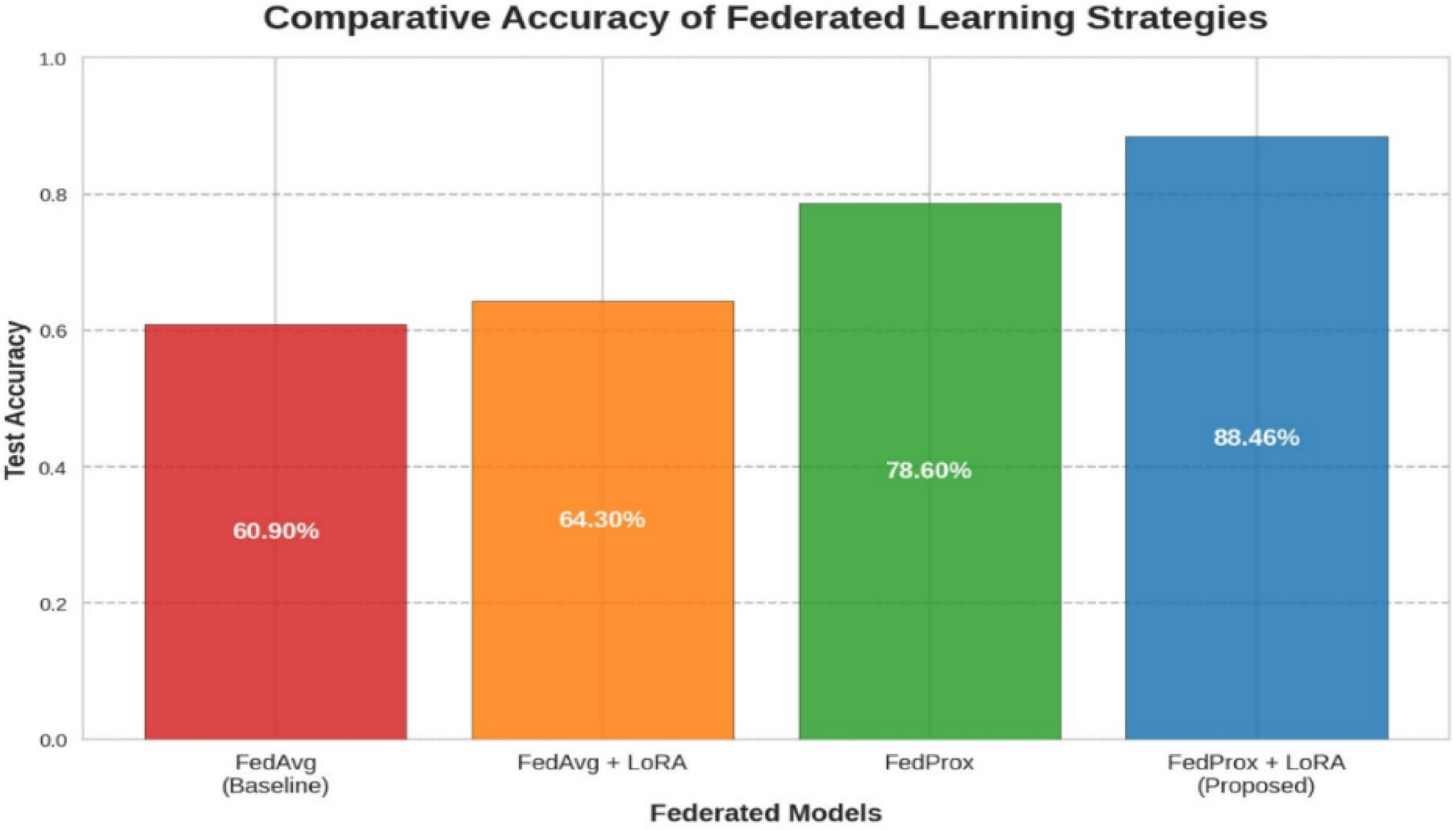
Accuracy of federated learning strategies.

**Fig. 11:**
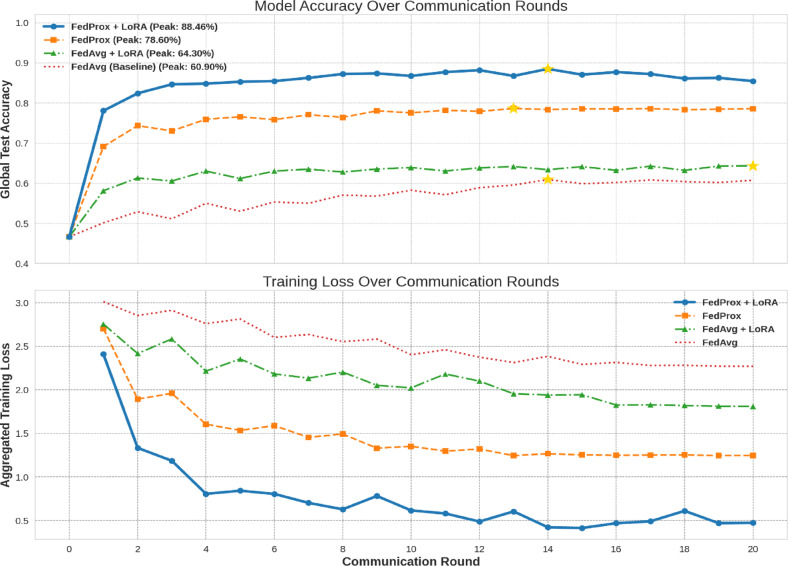
Convergence graph of federated strategies.

In order to evaluate the performance of the proposed federated model with FedProx strategy, an analysis is made with reference to accuracy and convergence profile. Figure 10 presents the comparison of accuracy of four FL strategies FedAvg, FedAvg + LoRA, FedProx, and FedProx + LoRA. Under client heterogeneity, the baseline FedAvg model has restricted accuracy of 60.90%. LoRA with FedAvg improves accuracy to 64.30% due to parameter-efficient fine-tuning. FedProx addresses data non-IIDness and boosts test accuracy to 78.60%. The FedProx + LoRA model performs best with an accuracy of 88.46%, exhibiting the synergistic benefits of stability and communication efficiency. This shows that robust optimization and adaptive tuning work in federated medical imaging applications. Figure 11 compares the convergence behavior and final accuracy of FedAvg, FedAvg + LoRA, FedProx, and FedProx + LoRA. This comparison shows that FedProx and LoRA improve training stability and communication efficiency in non-IID environments.

The comparative convergence profile shows four FL techniques’ performance and stability throughout 20 communication rounds. The FedProx + LoRA approach has the fastest convergence and highest peak test accuracy of 88.46%. FedProx-based methods beat FedAvg methods, demonstrating the algorithm’s ability to handle non-IID dispersed data. FedProx + LoRA consistently outperformed FedProx, showing a synergistic impact between robust aggregation and parameter-efficient updates.

**Fig. 12:**
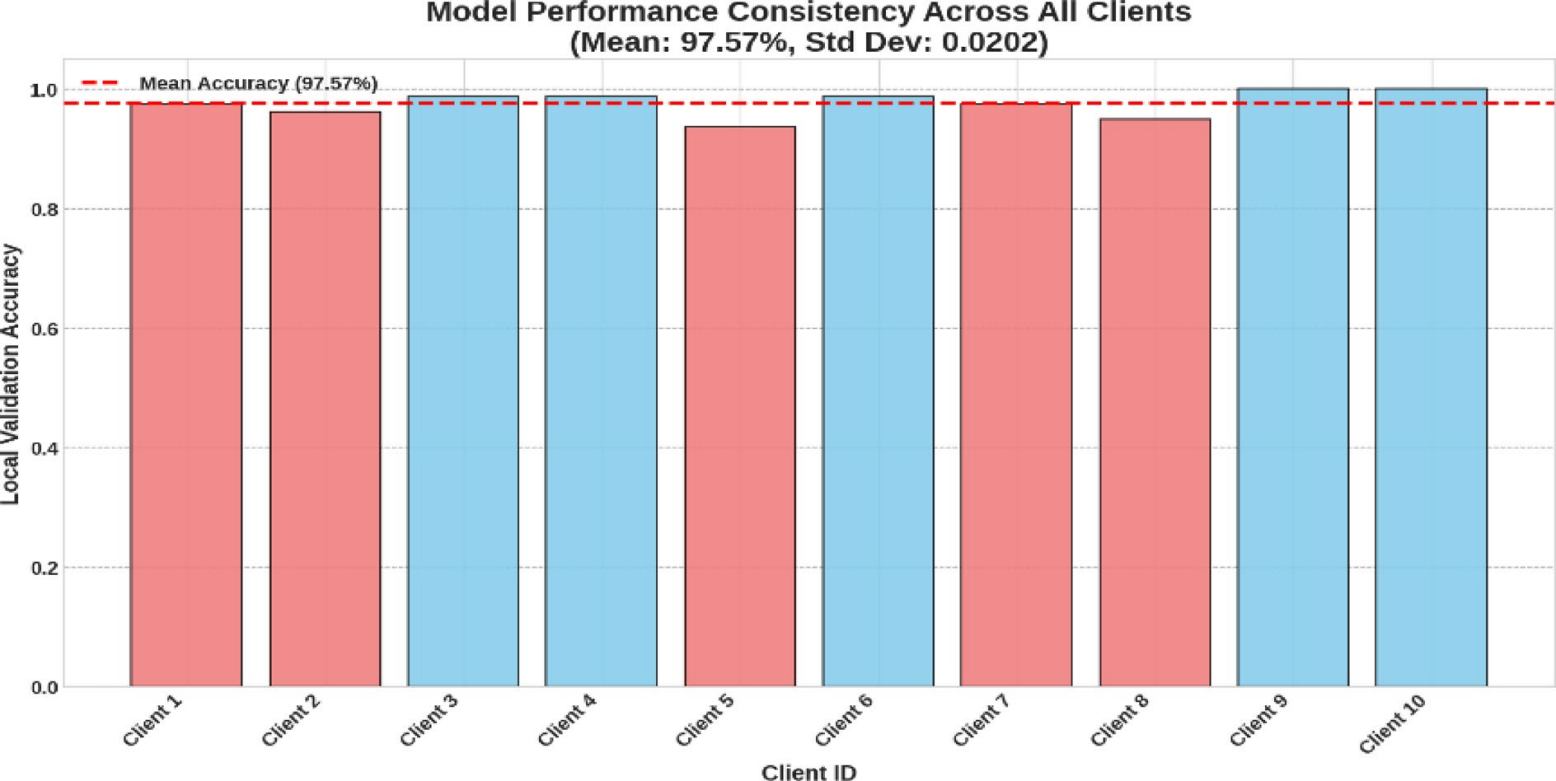
Local validation accuracy.

On the local validation set of each of the 10 clients as shown in Fig. 12, the final federated model is shown to be fair and resilient. The model had a mean accuracy of 97.57% and a standard deviation of 0.0202, demonstrating network-wide consistency. Even Client 5, the worst performer, had 93.59% accuracy, while two clients had 100%. This shows that the FedProx technique created a global model that generalizes effectively to each client’s data distribution, guaranteeing that collaborative training advantages are shared fairly.

### Explainability analysis

**Fig. 13:**
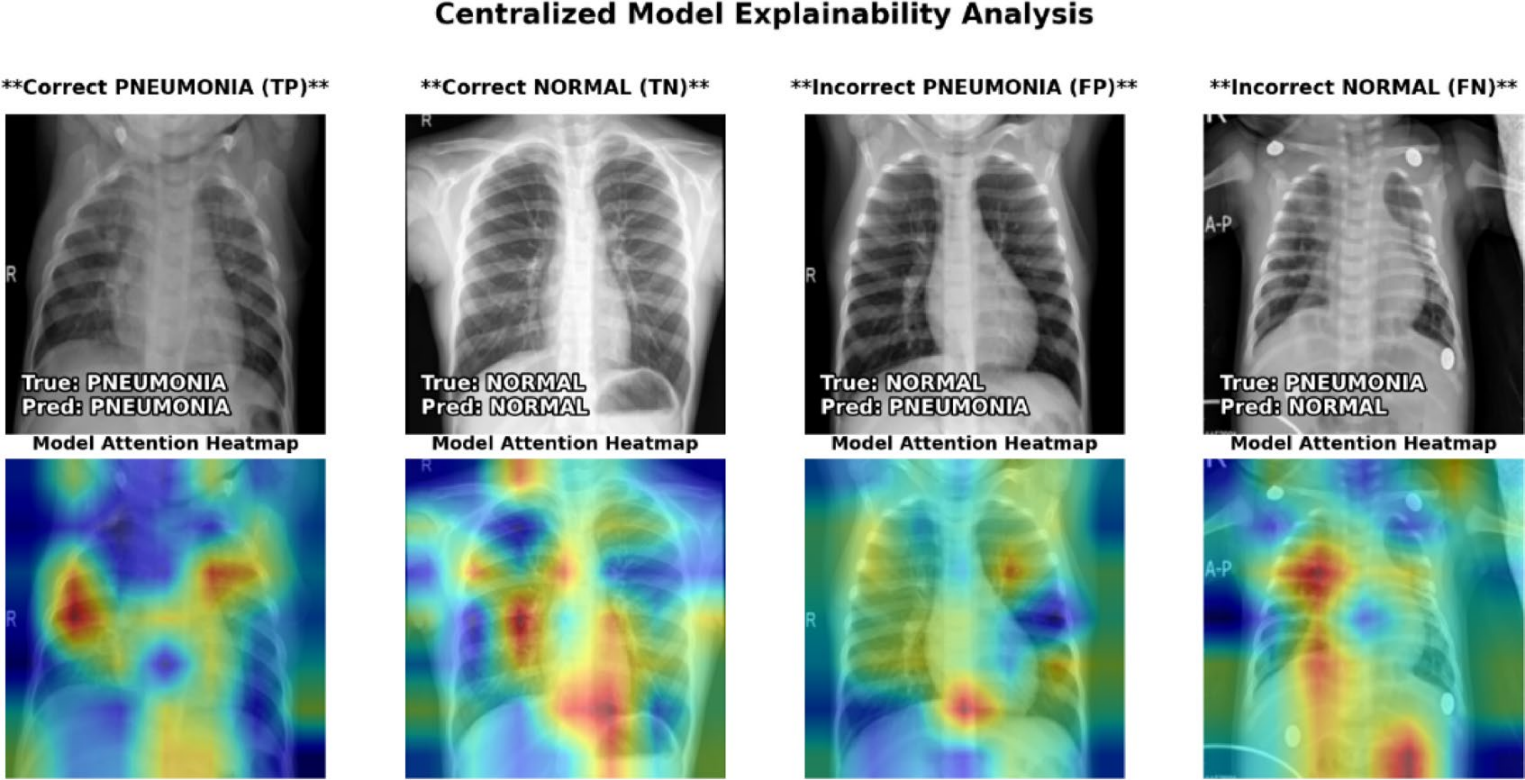
Centralized model explainability heatmaps.

**Fig. 14:**
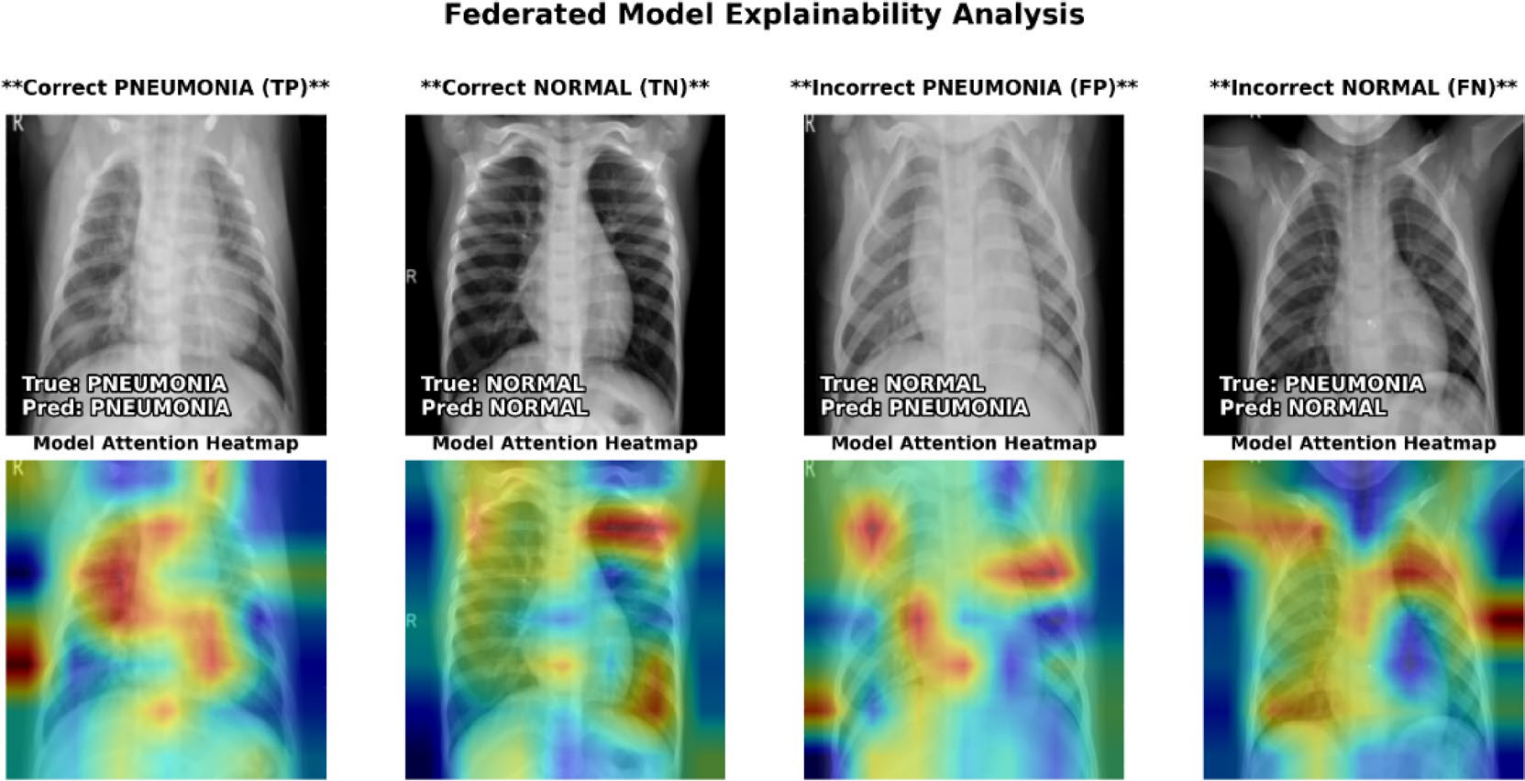
Proposed federated model explainability heatmaps.

Qualitative analysis of the centralized model’s test set predictions shows inconsistent and irrational behaviour. When predictions are wrong (False Positives and False Negatives), the model focuses on clinically unimportant artifacts rather than pathogenic indications, as shown in Figs. 13 and 14 as explainability heatmaps.

This visual evidence supports the poor quantitative metrics, demonstrating the model overfit to its training data and did not learn robust, generalizable characteristics needed for pneumonia diagnosis on out-of-distribution data.

**Fig. 15:**
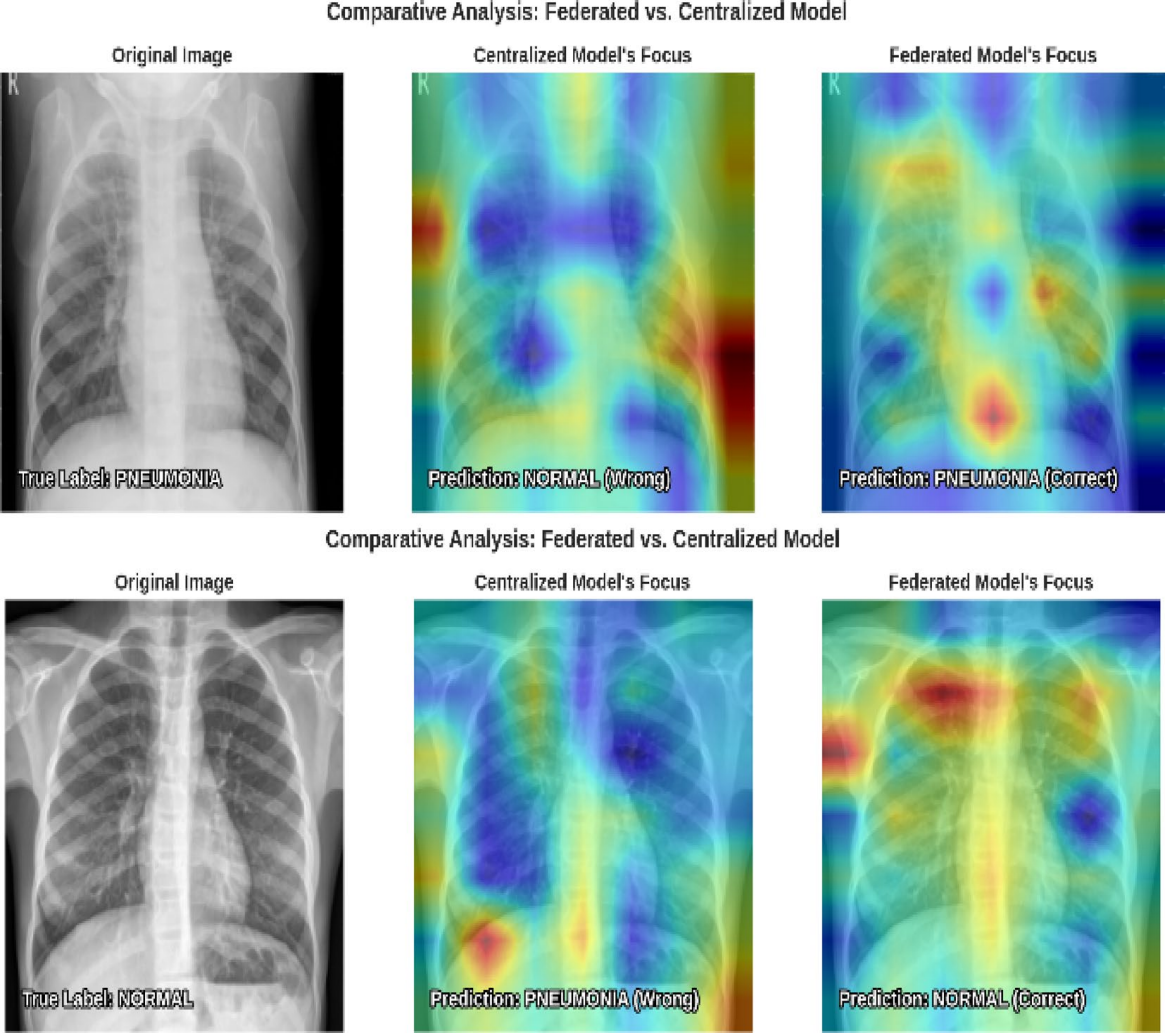
Sample predictions of centralized Vs federated analysis.

With an impressive 88.46% test accuracy and 0.96 AUC, the suggested federated model is a powerful diagnostic tool. The model’s clinical significance is shown by its 98.5% recall for PNEUMONIA, detecting 384 of 390 positive cases and missing just 6. This great quantitative performance is confirmed by qualitative analysis, where explainability heatmaps show that the model properly predicts lung pathogenic signs. In comparison analyses, the federated model accurately identified cases that perplexed the centralized model due to its capacity to generalize and focus on relevant features. A comparative analysis of sample of predictions made by centralized and federated models is shown in Fig. 15.

## Conclusion

A FL architecture is implemented for pneumonia detection utilizing chest X-ray images, using ViT as the backbone model, FedProx to address client heterogeneity, and LoRA for communication-efficient fine-tuning. The proposed model was tested in Non-IID data situations to simulate distributed, privacy-sensitive hospital patient data. It is observed that the proposed FedProx + LoRA-enhanced ViT model outperforms centralized and other federated baselines. The architecture is evaluated for classification efficiency with metrics accuracy, precision, recall, F1-score, AUC and federated efficiency based metrics convergence rate and communication cost. LoRA-based fine-tuning lowered model update size per round, making it suitable for bandwidth-constrained rural clinics. FedProx stabilized training across varied clients, speeding convergence and improving global model generalization, especially under healthcare-specific Non-IID data settings. In future, the algorithm can be extended to categorize various thoracic disorders beyond binary pneumonia detection utilizing larger datasets like CheXpert or MIMIC-CXR. This work provides the framework for privacy-preserving medical imaging diagnostic tools by integrating advanced transformer-based models with communication-efficient and robust federated techniques.

## Data Availability

The data used to support the findings of this study are available from the corresponding author upon request.
